# Work-related moderators of the relationship between organizational change and sickness absence: a longitudinal multilevel study

**DOI:** 10.1186/s12889-020-09325-w

**Published:** 2020-08-08

**Authors:** Anniken Grønstad, Lars Erik Kjekshus, Trond Tjerbo, Vilde Hoff Bernstrøm

**Affiliations:** 1 Department of Health Management and Health Economics, Institute of Health and Society, Faculty of Medicine, University of Oslo, Forskningsveien 3A, N-0373 Oslo, Norway; 2 Department of Sociology and Human Geography, Faculty of Social Sciences, University of Oslo, Moltke Moes vei 31, N-0851 Oslo, Norway; 3 Work Research Institute, OsloMet – Oslo Metropolitan University, Stensberggata 26, N-0170 Oslo, Norway

**Keywords:** Organizational change, Sickness absence, Moderation effects, Temporary contracts, Control, Organizational commitment, Hospitals, Norway

## Abstract

**Background:**

A sizeable body of research has demonstrated a relationship between organizational change and increased sickness absence. However, fewer studies have investigated what factors might mitigate this relationship. The aim of this study was to examine if and how the relationship between unit-level downsizing and sickness absence is moderated by three salient work factors: temporary contracts at the individual-level, and control and organizational commitment at the work-unit level.

**Methods:**

We investigated the association between unit-level downsizing, each moderator and both short- and long-term sickness absence in a large Norwegian hospital (*n* = 21,085) from 2011 to 2016. Data pertaining to unit-level downsizing and employee sickness absence were retrieved from objective hospital registers, and moderator variables were drawn from hospital registers (temporary contracts) and the annual work environment survey (control and organizational commitment). We conducted a longitudinal multilevel random effects regression analysis to estimate the odds of entering short- (< = 8 days) and long-term (> = 9 days) sickness absence for each individual employee.

**Results:**

The results showed a decreased risk of short-term sickness absence in the quarter before and an increased risk of short-term sickness absence in the quarter after unit-level downsizing. Temporary contracts and organizational commitment significantly moderated the relationship between unit-level downsizing in the next quarter and short-term sickness absence, demonstrating a steeper decline in short-term sickness absence for employees on temporary contracts and employees in high-commitment units. Additionally, control and organizational commitment moderated the relationship between unit-level downsizing and long-term sickness absence. Whereas employees in high-control work-units had a greater increase in long-term sickness absence in the change quarter, employees in low-commitment work-units had a higher risk of long-term sickness absence in the quarter after unit-level downsizing.

**Conclusions:**

The results from this study suggest that the relationship between unit-level downsizing and sickness absence varies according to the stage of change, and that work-related factors moderate this relationship, albeit in different directions. The identification of specific work-factors that moderate the adverse effects of change represents a hands-on foundation for managers and policy-makers to pursue healthy organizational change.

## Background

To provide excellent patientcare and contain costs, hospitals worldwide increasingly engage in organizational change. However, a sizeable pool of research has demonstrated associations between organizational change and adverse employee health and increased sickness absence [1, 2]. Despite the ramifications associated with sickness absence for organizations, employees, and society [3–6], organizational change remains a salient management strategy. Therefore, increased knowledge about work-related features that may increase or decrease the presence of sickness absence in times of organizational change is crucial.

Earlier research has often demonstrated increased sickness absence and reduced health related to both organization-level changes [2, 7] and unit-level changes [8–10]. For example, studies have found a relationship between unit-level structural changes [9], and organizational-level merger [2], and increased probability of > 16 days sickness absence [2, 9]; between unit-level merger and unit-level layoffs and increased risk of > 28 days absence spells [10], and between organizational-level downsizing and increased risk of > 3 days absence spells [7]. However, noteworthy studies have also found limited and even lower odds of long-term sickness absence (> = 15 and > =4 days) in relation to downsizing [11, 12].

Such contradictory findings could be a result of different employee experiences of similar changes [13]. For example, merger is a type of change that may influence employee health status both positively and negatively [14]. Therefore, identifying work-related moderators is an important step toward elucidating how workplace factors may influence employee sickness absence in times of organizational change. Additionally, increased knowledge about potential moderators will help managers and practitioners to remedy the adverse effects and promote the beneficial effects of organizational change. In a recent review, Oreg et al. [15] concluded that identifying moderating variables is essential to enhance our understanding of change recipients’ reactions to change. However, few empirical studies have measured such moderation effects [13, 15].

The objective of the present paper is to examine if and how three work factors - temporary contract, and work environment measures for control and organizational commitment at the unit-level, moderate the relationship between unit-level downsizing and sickness absence in a large Norwegian hospital. In a previous study, we identified unit-level downsizing as a frequently occurring type of unit-level change related to both decreasing and increasing odds of short-term sickness, depending on the stages of change [16]. Specifically, unit-level downsizing was associated with reduced short-term sickness absence prior to the change and increased short-term sickness absence during and after unit-level downsizing. The prevalence and potential ramifications of unit-level downsizing have motivated a further examination of this particular change type.

In this paper, we define unit-level downsizing as a > 20% staff reduction in a work-unit during a quarter. Within the hospital, unit-level downsizing rarely involves layoffs. Instead, units become smaller by not renewing temporary contracts, not filling vacant positions, and offering employees work elsewhere within the hospital or nearby hospitals. Such strategies for downsizing without layoffs are similar to those employed for downsizing at the organizational level [17].

In this paper, we investigate both short- and long-term sickness absence, because the causes may differ between them. Long-term and medically certified sickness absence are often viewed as valid and strong indicators of employee health [6, 18]. Short-term sickness absence, however, is more prone to be affected by work-factors, also when health is controlled for [18, 19], and may therefore also reflect a motivational component to attend work. Earlier research has defined short- and long-term sickness absence in several different ways. For example, short-term sickness has been defined as periods lasting up to 3 days [7, 20], up to 7 days [21], and up to 42 days [22]. Similarly, long-term sickness absence has been defined as absence periods lasting more than 3 days [7, 20], more than 16 days [2, 9], more than 28 days [10], more than 55 days [23], and more than 90 days [24]. In Norway, public hospital employees have the authority to self-certify sickness absence periods lasting 8 days or less. From the 9th day onwards, the employees need to obtain a medical sickness absence certificate issued by their GP. In the current paper, we have defined short- and long-term sickness absence as < = 8 days and > = 9 days, respectively because a medical certificate is required from the 9th day of absence, making it a meaningful cut-off point in the current context. Separating lengths of sickness absence based on the specific research context has also been applied elsewhere [21, 25].

## Moderators of the relationship between organizational change and employee health

Few earlier studies on organizational change and sickness absence have included moderators. However, certain studies have demonstrated that the positive relationship between downsizing and long-term sickness absence is stronger among older employees, employees with higher income, employees working in larger workplaces with a higher proportion of older employees and employees with permanent job contracts [20, 26].

In this paper, we focus on three organizational factors that we expect will moderate the association between unit-level downsizing and sickness absence: temporary contracts, and work environment measures for control and organizational commitment at the unit-level.

### Temporary contracts

The first moderator we investigate is whether the employees had a temporary contract and how the presence of temporary contracts may influence the association between unit-level downsizing and sickness absence. Temporary contracts may influence employee attendance motivation through economic pressures if employees attend work despite illness due to fear of not having their contracts renewed [27]. Indeed, employees on temporary contracts have shown lower medically certified sickness absence (> 3 days) [28] and Vahtera et al. [26] supported the moderating effect of temporary contracts, demonstrating stronger increases in medically certified absence spells among permanent employees, compared to temporary employees, in times of downsizing.

In times of unit-level downsizing, pressures to maintain a good attendance rate may increase because employees increasingly worry about potential job loss. Because ceasing to renew temporary contracts is a common downsizing-strategy, downsizing will likely foster a steeper rise in job insecurity for temporary employees compared to permanent employees. We therefore expect unit-level downsizing to have a more profound effect on temporary employees’ attendance motivation compared to permanent employees. Consistent with earlier literature, we expect that temporary contracts will moderate the relationship between unit-level downsizing and sickness absence. Specifically, we expect that any increase in sickness absence will be weaker among temporary employees and that any decrease in sickness absence will be steeper among temporary employees, compared to permanent employees.

### Control in the work-units

The second moderator we investigate is the level of employee control in their work-units. Often referring to the job demands-control model [29, 30], numerous studies have demonstrated that job control is an important work-related factor influencing employee health [31–33]. Indeed, earlier research has identified associations between low control and increased risk of sickness absence [34–36], also in the context of major downsizing [37]. In a related vein, health benefits emerge and adverse health effects of workplace stress decline when perceived control is good or improved [38, 39]. Based on earlier literature emphasizing the ameliorating effect of enhanced control, we may speculate that high control acts as a resource boosting employee capacity to handle possibly straining situations.

The general buffering potential of control has received mixed support. Indeed, not all studies support the interaction effect between demands and control [32, 40, 41]. However, studies focusing on specific change-related issues support the moderating effect of control on the relationship between change-related job insecurity and adverse health outcomes [42, 43]. These studies propose that the adverse health effects of job insecurity can be attenuated by high levels of control.

In this paper, we expect that control in the work-units will moderate the relationship between unit-level downsizing and sickness absence. Specifically, we expect that any increase in sickness absence will be weaker for employees in work-units with high levels of control.

### Organizational commitment in the work-units

The third moderator we investigate is employees’ level of commitment to the organization in their work-units. Commitment to the organization can act as a moderating variable through both employee motivation and employee ability to attend work [27]. According to Steers and Rhodes [27], organizational commitment fosters an alignment with the organization’s goals. By firmly believing in the organization’s purpose, employees are expected to have a stronger attendance motivation and to contribute to realize the goals. In that regard, earlier research has observed an association between higher commitment and lower absenteeism [44]. Such motivating pressures remain regardless of whether or not employees appreciate all aspects of work [27]. We therefore expect that employees who are highly committed to the organization, to a greater extent than less-committed employees, will consider attending work in times of organizational change to be particularly important in order to contribute to the change process and protect valued aspects of work. Moreover, highly committed employees may also, to a lesser extent than less-committed employees, allow negative sentiments regarding the change process to influence their attendance motivation. Work-units comprising highly committed employees will thus stimulate a stronger attendance motivation among their members. In support of such arguments, Begley and Czajka [45] identified commitment as a moderator in the context of organizational turmoil, demonstrating that low-committed employees had a significant increase in job displeasure as stress increased, whereas high-committed employees displayed no such effects.

However, organizational commitment might also influence absence levels via employee health (i.e. the ability to go to work). In this regard, commitment might play a dual role. On the one hand, highly committed employees may become more disturbed and stressed by challenges the organization encounters owing to the close alignment of the employees’ and the organization’s goals [46], and thus be more vulnerable and sensitive to harmful consequences of organizational change. On the other hand, organizational commitment may act as a resource that protects employees from the undesirable effects of change-related stress. For example, Mowday et al. [47] argued that organizational commitment might foster employees’ feelings of belongingness, security, and stability, enable employees to attribute meaningfulness and direction to their work situation [47] and to avoid symptoms of strain [48]. Hence, organizational commitment may encapsulate resources that are available to highly committed employees possibly influencing how they appraise change scenarios and cope with situations they appraise as threatening [49]. As downsizing, arguably, can be viewed as a taxing type of change, it is possible that working in a high-commitment unit enhances employees’ coping resources and thus protects them from the potential adverse health effects that may emerge from downsizing.

Based on these arguments, we expect that organizational commitment in work-units will moderate the relationship between unit-level downsizing and sickness absence. Specifically, we expect that any increase in sickness absence will be weaker among employees working in a unit characterized by high commitment, and that any decrease in sickness absence will be steeper among employees working in a unit characterized by high commitment.

## Methods

### Participants

#### The hospital

We analyzed data that were retrieved from a large Norwegian hospital from 2011 to 2016. The hospital employs more than 20,000 workers dispersed at several different locations. The hospital had 1512 work units, employing between one and 168 employees each (20 employees on average). In addition to providing high-quality healthcare, the hospital also engages in research and has a responsibility for medical education and training. The hospital is highly specialized and has local, regional, and nationwide patient-treatment responsibility.

#### The employees

The data contained 27,468 unique employee ID numbers for the given study period. In the final sample, we included fulltime employees only (> = 80% cumulative employment contracts) who worked in units comprising a minimum of four employees. To compare downsizing to stability, quarters in which employees experienced other organizational changes (i.e. spin-off, merger, upsizing, insourcing, and outsourcing at the unit-level [16]) were dropped from the analyses.

### Data and measures

We employed two different sources of merged data drawn from registers in the hospital’s Human Resources (HR) department, in addition to the hospital’s work environment survey. Using HR register data allowed us to track unit-level downsizing and sickness absence over several years for the same individual employees, using objective data with a high level of accuracy. Although the hospital continuously recorded the HR registries, we aggregated the data into employee-quarters to make them suitable for analysis. By using the hospital’s annual work environment survey, aggregated to the unit-level, we could also track each work-unit’s measures for control and commitment for the same period.

In this manner, the data were organized as employee-quarters (i.e. our level of observation), nested within employees, nested within work units. Because we did not have work environment data for 2014 and 2016, registry information from these years was only utilized when relevant for adjacent quarters (e.g. registry data from Q4 2014 provide information on employees who went through “downsizing in the last quarter” in Q1 2015). Similarly, because we did not have information on whether employees in Q1 2011 experienced downsizing in the previous quarter, Q1 2011 was dropped from the analyses.

The final sample consisted of 173,787 observations, nested in 21,085 employees nested in 1167 work-units. Each employee was observed between one and 15 quarters, and eight on average. Each work unit consists of between one and 114 employees each, and 18 on average.

The project was reported to the Norwegian Data Protection Authorities (NSD) and data protection officers at the hospital and complied with the Declaration of Helsinki.

#### Sickness absence

We used data on employee sickness absence retrieved from hospital records. Within these registers, all absence periods were listed with the beginning and end date as well as the percentage of absence. We included all registered sickness absence, irrespective of percentage, in the analyses. Consecutive absence periods were merged. Registering sickness absence is a system-based prerequisite for the work-units to recruit substitute personnel. These objective records should therefore be regarded as highly accurate and complete compared to alternative measures such as self-reported absence. The dependent variable in this study was whether an employee entered a period of sickness absence during the quarter. Because shorter spells are more likely to be influenced by aspects other than health [18, 19], we aggregated the data to separate short- (<=8 days) and long (> = 9 days) periods of sickness absence. For absences lasting 9 days or longer, the employee is required to obtain a medical sickness absence certificate. Because the present study was carried out in the Norwegian hospital sector context, we decided to separate between short-term (1–8 days) and long-term (> = 9) sickness absence.

#### Unit-level downsizing

We derived data on downsizing from hospital records of employment contracts. These registers provided historical data regarding employees’ contracts throughout the study period. The particular change was identified by tracking the specific units in which the employees worked each quarter and how they shifted units. We quantitatively operationalized unit-level downsizing by adopting the threshold defined by Røed and Fevang [17]: a major change necessitates a reduction in employees amounting to more than 20%. In this study, this meant that if a unit downsized by > 20% in one quarter (without simultaneously experiencing an outsourcing amounting to > 90% of the unit or a unit-level merger), we coded the employees as being downsized. The change variable was binary, given values of 0 (stability) or 1 (unit-level downsizing). Measured quarterly, we identified whether an employee was anticipating downsizing in the upcoming quarter (i.e. “unit-level downsizing next quarter”), presently experiencing downsizing (i.e. “unit-level downsizing this quarter”), had experienced downsizing in the previous quarter (i.e. “unit-level downsizing previous quarter”) or was having stability (i.e. the control group). In this paper, experiencing stability entailed an absence of unit-level downsizing, in addition to the absence of unit-level spin-off, merging, upsizing, insourcing and outsourcing of units [16].

#### Temporary contracts

The data on temporary contracts were drawn from HR registers on employment contracts. In the register, employment contract was categorized as being either temporary or permanent. The objective registers could therefore be considered as highly accurate and complete.

#### Work environment moderators

The final source of data was employees’ experiences as reported in the work environment survey. This was a comprehensive survey that the hospital conducted in 2011 (response rate 70%), 2012 (response rate 72%), 2013 (response rate 80%), and 2015 (response rate 76%).

The measures for control and organizational commitment were developed and validated by the General Nordic questionnaire (QPS Nordic) for psychological and social factors at work [50]. Both variables were measured using self-report on a 5-point Likert scale. For control, the scale ranged from “very seldom or never,” coded as (0), to “very often or always,” coded as (4). For commitment, the response scale ranged from “disagree totally,” coded as (0) to “agree totally,” coded as (4). The sample items used to measure control at work included “Can you set your own work pace?” and “Can you influence the amount of work assigned to you?” [50]. The sample items measuring organizational commitment from this scale included “This organization really inspires me to give my very best job performance” and “To my friends, I praise this organization a great place to work” [50]. The Cronbach’s alpha was 0.795 for control and 0.873 for organizational commitment.

We aggregated the work environment factors to the work-unit level before merging them with the rest of the data. The mean values of each specific work-unit were assigned to all of the employees working in that particular unit. Each employee-quarter was given the value from the employees’ work-unit that specific year. Aggregating the work environment factors to unit-level was necessary. Anonymity concerns only permitted us to identify the specific work-units in which the employees worked, as opposed to identifying each individual respondent. Although aggregation necessarily meant that some data were lost, it was also an advantage because it allowed us to focus on the work-units’ work environment, rather than on individual perceptions. As argued by Hausknecht et al. [44], conceptualizations of sickness absence have often focused on individual-level predictors, thus diminishing the work-unit context that frames sickness absence behavior.

When aggregating the work environment survey, it was also important to consider group agreement. Commitment to the organization demonstrated strong agreement within the work-units with an r_WG_ of 0.73. Control showed only moderate agreement with an r_WG_ of 0.64 [51]. Traditionally, 0.7 has been set as a cut-off point denoting higher interrater agreement and acceptable aggregation. Lebreton and Senter [51] argued that this cut-off point might be too high in some instances (e.g., the measure is not used for decisions involving specific individuals). Because we could only merge the work environment variables with the rest of the data at the work-unit level, we also aggregated control. However, the moderate level of agreement needed to be taken into consideration when interpreting the findings. Expectation-Maximization algorithm (EM) was used for missing values.

#### Control variables

Several variables likely to influence the dependent variable were drawn from the HR registers on employment contracts, and were included as control variables. In this paper, control variables comprised salary, age, gender, country of origin, job position, and multiple jobs. We operationalized country of origin into Norwegian, other Nordic, other Western and non-Western. We coded job position into physician, nurse, other patient-oriented position, administration/management, kitchen/cleaning/orderly, other operations (e.g. IT), and other*.* As hospital employees commonly hold more than one job contract, we included a dummy variable to specify when an employee was a multiple job-holder. Age was measured in years and salary comprised contracted remuneration. Gender was dummy coded (female =1, male = 0). In addition, all three potential moderating variables are also included as control variables, because they are also likely to be confounding variables (e.g. if units with a high percentage of temporary contracts are more likely to downsize).

### Statistical analysis

We used a multilevel regression model with random intercept at employee- and work-unit level to analyze the relationship between unit-level downsizing, moderators (temporary contract, control and organizational commitment, separately) and sickness absence. Hence, the unit of analysis was employee-quarters nested within employee, and employees nested within work-unit. For the purpose of analysis, we hierarchically nested employees according to the unit in which they most often appeared. In all, 81% of employees appeared in the same unit in all observations. Stress testing the findings by excluding all quarters in which the employees did not work in their main unit did not significantly alter the results.

We ran a multilevel model because such models are suitable to account for dependency in the data (i.e. employees measured at multiple time points are more similar to themselves than to others, and employees within one work-unit are more similar to each other compared to employees at other units). Multilevel models explicitly and properly estimate the degree of dependencies of observations (i.e., non-independence), thereby protecting researchers from an error of inference and spurious significant results [52–54]. Moreover, random effects regression offers the most efficient estimator of the relationship between unit-level downsizing and sickness absence by measuring both the variation between employees and within employees, in a longitudinal design [55]. The dependent variable (sickness absence) was examined at the lowest level of analysis (i.e., employee-quarters). The dependent variable, episodes of sickness absence, was binary; therefore, we used multilevel logistic regression.

We ran separate analyses for each of the three moderators (temporary contract, work-unit control and work-unit organizational commitment). We estimated moderating effects by testing for significant interactions between unit-level downsizing and each moderator, respectively [56]. The level of statistical significance was set to *P* < .05. All analyses were performed using the xtmelogit command in STATA/SE, version 15.1 (StataCorp LP, College Station, TX, USA, http:www.stata.com/company/contract/).

## Results

Employee descriptive statistics are detailed in Table 1. The hospital workforce included a multitude of professions such as physicians, nurses, engineers, economists, laundry service, and kitchen service. The majority of the employees were female (74%) and were Norwegian in origin (93%). Throughout the study period, 79% of the employees at the hospital had at least one period of short-term sickness absence. By contrast, 44% of the hospital employees had at minimum one incidence of long-term sickness absence. During the study period, 19% of employees experienced anticipating downsizing (“i.e. downsizing in the next quarter) at least once.

**Table 1:** Descriptive statistics

	Number of employees	% of N (employees)
N	21,085	
Unit-level downsizing next quarter	4037	19%
Unit-level downsizing this quarter	3805	18%
Unit-level downsizing previous quarter	3245	15%
Short-term sickness absence	16,612	79%
Long-term sickness absence	9178	44%
Temporary contract	8101	38%
Unit-level commitment	1,8	(SD 0.6 Range 0–4)
Unit-level control	2,8	(SD 0.4 Range 0–4)
Age	44	(SD 12)
Salary	529	(SD 227)
Female	15,535	74%
Physician	3043	14%
Nurse (control)	7450	35%
Other patient-related position	4987	24%
Administration/management	3068	15%
Kitchen/cleaning/orderly	842	4%
Other operations	870	4%
Other	825	4%
Multiple job holder	5170	25%
Norwegian (control)	19,513	93%
Other Nordic	809	4%
Other Western	400	2%
Non-Western	363	2%

Descriptive statistics for unit-level downsizing, temporary contract and short- and long-term sickness absence show the percentage of employees with minimum one instance on the given variable. Salary, age, and unit-level commitment and control are given as employees’ mean throughout the study period. N refers to all employees qualified to be included in the analyses

Table 2 presents the association between unit-level downsizing and sickness absence, including Model 1 without control variables and Model 2 with control variables. For the purpose of interpretation, we place emphasis on Model 2. The results showed that the risk of short-term sickness absence significantly decreased in the quarter preceding unit-level downsizing (OR = 0.68; CI = 0.63–0.73) and significantly increased in the quarter during and after unit-level downsizing (OR = 1.08; CI = 1.00–1.17; OR = 1.24; CI = 1.14–1.35, respectively). No significant effects were observed for the relationship between unit-level downsizing and long-term sickness absence.

**Table 2:** Associations between unit-level downsizing and sickness absence

Change type	Model 1^**a**^		Model 2^**b**^	
	Short-term SA	Long-term SA	Short-term SA	Long-term SA
	(< = 8 days)	(> = 9 days)	(< = 8 days)	(> = 9 days)
	OR 95% CI	OR 95% CI	OR 95% CI	OR 95% CI
Unit-level downsizing next quarter	0,67 (0,62-0,72)***	0,88 (0,78-0,98)**	0,68 (0,63-0,73)***	0,93 (0,83-1,04)ns
Unit-level downsizing this quarter	1,07 (0,99-1,16)ns	0,95 (0,85-1,06)ns	1,08 (1,00-1,17)*	0,99 (0,89-1,10)ns
Unit-level downsizing previous quarter	1,22 (1,12-1,33)***	1,05 (0,93-1,18)ns	1,24(1,14-1,35)***	1,08 (0,96-1,22)ns
Temporary contract			0,97 (0,92-1,01)ns	0,65 (0,61-0,70)***
Unit-level comittment			0,95 (0,91-0,99)**	0,87 (0,83-0,92)***
Unit-level control			0,92 (0,88-0,97)***	0,93 (0,89-0,98)**
Salary			1,00 (1,00-1,00)***	1,00 (1,00-1,00)***
Female			1,26 (1,19-1,33)***	1,94 (1,82-2,07)***
Age			0,99 (0,99-0,99)***	1,01 (1,00-1,01)***
Multiple job holder			1,12 (1,06-1,19)***	1,16 (1,07-1,25)***
Nurse (control)				
Physician			0,40 (0,35-0,44)***	0,65 (0,58-0,74)***
Other patient-related position			0,90 (0,83-0,96)**	1,00 (0,92-1,07)ns
Administration/management			0,63 (0,58-0,69)***	0,83 (0,77-0,91)***
Kitchen/cleaning/orderly			1,80 (1,52-2,14)***	2,48 (2,14-2,87)***
Other operations			0,76 (0,67-0,86)***	1,11 (0,96-1,27)ns
Other			0,20 (0,17-0,24)***	0,52 (0,43-0,63)***
Norwegian (control)				
Other Nordic			1,16 (1,04-1,29)**	0,89 (0,78-1,01)ns
Other Western			0,84 (0,72-0,99)*	0,85 (0,69-1,04)ns
Non-Western			0,90 (0,76-1,07)ns	0,86 (0,70-1,06)ns

Logistic regression. Random effects analyses: random intercept for employee and work-unit

*Abbreviations*: *SA* sickness absence, *OR* odds ratio, *CI* 95% confidence intervals, *ns* not significant

Significance

* *p* < .05

** *p* < .01

*** *p* < .001

^a^Control variables not included

^b^Control variables included

N-work-units: 1167; N-employees: 21,085; N-observations: 173,787

The results of the multilevel regression models, including the interaction effects of temporary contracts, control, and organizational commitment, are presented in Table 3. For the results without control variables and results including all interactions in the same analysis, please see Additional file 1 (Table A). The results in Table A are similar to those presented in Table 3. Therefore, emphasis is placed on Table 3 when interpreting the results.

**Table 3:** Associations between unit-level downsizing and sickness absence – moderation effects^a^

	Short-term SA	Long-term SA
	(< = 8 days)	(> = 9 days)
	OR 95% CI	OR 95% CI
Unit-level downsizing next quarter	0,74 (0,68-0,81)***	0,94 (0,83-1,07) ns
Unit-level downsizing this quarter	1,04 (0,95-1,14) ns	0,96 (0,85-1,08) ns
Unit-level downsizing previous quarter	1,21 (1,10-1,33)***	1,12 (0,99-1,27) ns
Temporary contract (TC) (scale: 0–1)	0,97 (0,92-1,02) ns	0,66 (0,61-0,70)***
Unit-level downsizing next quarter X TC	0,75 (0,63-0,89)**	0,93 (0,71-1,21) ns
Unit-level downsizing this quarter X TC	1,13 (0,95-1,34) ns	1,17 (0,89-1,52) ns
Unit-level downsizing previous quarter X TC	1,09 (0,90-1,33) ns	0,81 (0,58-1,12) ns
Unit-level downsizing next quarter	0,55 (0,43-0,70)***	0,83 (0,57-1,20) ns
Unit-level downsizing this quarter	1,19 (0,93-1,50) ns	0,68 (0,47-0,97)*
Unit-level downsizing previous quarter	1,32 (1,02-1,71)**	1,24 (0,86-1,80) ns
Control (scale: 0–4)	0,92 (0,88-0,97)**	0,93 (0,88-0,98)**
Unit-level downsizing next quarter X control	1,13 (0,99-1,29) ns	1,06 (0,88-1,29) ns
Unit-level downsizing this quarter X control	0,95 (0,84-1,08) ns	1,23 (1,02-1,49)*
Unit-level downsizing previous quarter X control	0,96 (0,84-1,10) ns	0,93 (0,76-1,13) ns
Unit-level downsizing next quarter	1,09 (0,72-1,65) ns	0,95 (0,53-1,70) ns
Unit-level downsizing this quarter	0,97 (0,65-1,45) ns	0,66 (0,37-1,18) ns
Unit-level downsizing previous quarter	1,10 (0,71-1,70) ns	2,11 (1,18-3,78)*
Organizational commitment (OC) (scale: 0–4)	0,95 (0,91-0,99)*	0,88 (0,83-0,92)***
Unit-level downsizing next quarter X OC	0,84 (0,73-0,98)*	0,99 (0,80-1,23) ns
Unit-level downsizing this quarter X OC	1,04 (0,90-1,20) ns	1,16 (0,94-1,42) ns
Unit-level downsizing previous quarter X OC	1,04 (0,89-1,22) ns	0,78 (0,64-0,97)*

Logistic regression. Random effects analyses: random intercept for work-unit

*Abbreviations*: *SA* sickness absence, *OR* odds ratio, *CI* 95% confidence intervals, *ns* not significant, *TC* temporary contracts, *OC* organizational commitment

Significance

* *p* < .05

** *p* < .01

*** *p* < .001

^a^Control variables included (temporary contract, control, organizational commitment, salary, gender, age, multiple job holder, position, nationality)

N-work-units: 1167; N-employees: 21,085; N-observations: 173,787

The results revealed two significant moderators for the relationship between unit-level downsizing and short-term sickness absence. There was a significant negative interaction between temporary contracts and unit-level downsizing in the next quarter, demonstrating that the decline in odds of short-term sickness absence was greater for employees on temporary contracts (OR = 0.75; CI = 0.63–0.89). Similarly, there was a negative interaction between organizational commitment and unit-level downsizing in the next quarter for short-term sickness absence, demonstrating that the decline in odds of short-term sickness absence was greater for employees in units with high organizational commitment (OR = 0.84; CI = 0.73–0.98). Moreover, the results showed no significant change in short-term sickness absence for employees working in units characterized by low levels of organizational commitment. However, further inspection of the numbers revealed that employees who worked in a unit with an average level of organizational commitment of three or above (on a Likert scale ranging from 1 to 5) had a significantly reduced risk of short-term sickness absence in the quarter prior to unit-level downsizing. In total, 99% of the employees worked in a unit with an average level of organizational commitment of three or more.

The results further revealed two significant moderators for the relationship between unit-level downsizing and long-term sickness absence. The relationship between unit-level downsizing this quarter and long-term sickness absence was moderated by control. Further inspection of the numbers revealed that employees in work-units with high levels of control (on average four or five on a 1–5 Likert scale, approximately 15% of the sample) had a significantly increased risk of long-term sickness absence in the change quarter (OR = 1.23; CI = 1.02–1.49). No such effect was found for employees in work-units with lower levels of control. Rather, the results indicated that employees working in units with no control (approximately 0.5% of the sample) had a significant reduction in long-term sickness absence in the change quarter.

Lastly, the results also showed a significant interaction effect between unit-level downsizing in the previous quarter and organizational commitment. The results demonstrated an increase in long-term sickness absence during the quarter for employees in work-units with low organizational commitment. The increase in long-term sickness absence lessened as organizational commitment increased (OR = 0.78; CI = 0.64–0.97). However, closer inspection of the numbers showed that the increased risk of long-term sickness absence remained significant for employees in units with an average level of organizational commitment of three or lower (approximately 20% of the sample). In other words, unit-level downsizing in the previous quarter was significantly related to increased long-term sickness absence for employees in units where they on average replied “disagree totally,” “disagree to some extent,” or “indifferent” to statements such as “To my friends, I praise this organization a great place to work”.

All analyses were initially run without control variables. Including control variables resulted in one non-significant interaction term: the moderating effect of temporary contracts on the relationship between unit-level downsizing in the previous quarter and long-term sickness absence.

## Discussion

In this paper, we examined the association between unit-level downsizing and sickness absence, with a particular focus on the moderating effects of temporary contracts, and control and organizational commitment at the unit-level.

The results of this study show a significantly reduced risk of short-term sickness absence prior to unit-level downsizing and a significantly increased risk of short-term sickness absence after unit-level downsizing. Similar to our previous study [16], the results only partially corroborate prior research showing a relationship between organizational change and increased sickness absence and reduced health [2, 57], also at the unit-level [8–10]. Indeed, in line with Østhus and Mastekaasa [12], our results did not show increased long-term sickness absence at any stage of the downsizing process.

To explain reduced sickness absence levels associated with downsizing, earlier research has suggested that employees experiencing downsizing are hesitant about being absent due to increased job insecurity [11, 26]. The results from the moderation analyses in this study partially support this argument. Indeed, the results of the first moderation analysis support our expectations that employees on temporary contracts display a stronger reduction in the odds of short-term sickness absence prior to unit-level downsizing. This is partly in line with Vahtera et al. [26] who observed that downsizing was associated with a rise in sickness absence among those who were permanently employed but not among temporary employees. Not renewing temporary contracts is a common downsizing-strategy in Norwegian hospitals. Arguably, because temporary employees are more likely to lose their jobs during downsizing, increased perceptions of job insecurity may encourage them to attend work while ill [58, 59]. In other words, we may speculate that economic pressures [27] stemming from worrying about contract renewal might create disciplinary incentives for ill employees to attend work. Such an understanding supports the interpretation that reduced absence levels prior to unit-level downsizing indeed reflect a change in attendance behavior due to increased job insecurity.

Noticeably, however, the moderation analyses also show reduced short-term sickness absence for permanent employees in the quarter prior to downsizing (albeit to a lesser extent). As termination of contracts is very rare in Norwegian hospitals, reduced short-term sickness absence among permanent employees is difficult to explain in terms of fear of job-loss.

In contrast to our expectations, no significant moderation effects were observed for temporary contracts on the relationship between unit-level downsizing this quarter or last quarter and sickness absence. A potential explanation for these results may be that employees receive a notice in advance, of whether their contracts will be renewed. Once a decision has been made, the disciplinary effects subside.

The results of this study further support our expectations that employees working in units with high levels of commitment have a stronger reduction in the odds of short-term sickness absence prior to unit-level downsizing, compared to employees in low-commitment units. While temporary contracts may foster reductions in sickness absence due to disciplinary attendance motivation, organizational commitment might be a more positive driver of attendance. Employees working in high-commitment units generally have lower sickness absence than employees in units with lower levels of commitment. As with temporary contracts, this difference grows in the period prior to unit-level downsizing. Employees working in high-commitment units might feel a greater need to be present for their work and patients in times of turmoil. Additionally, employees who are more committed to their organization might also have more at stake as things change. A higher degree of presence prior to unit-level downsizing might therefore also reflect employees’ aspiration to participate in the change process and protect valued aspects of work, such as different roles and tasks. Furthermore, when a unit generally has high levels of commitment, the work environment may encourage participation and attendance in times of change, thus allowing employees to influence the direction of the change in a more desired way.

The results also showed that employees in units with low commitment to the organization had significantly increased long-term sickness absence in the quarter following unit-level downsizing. These different reactions to change according to the amount of commitment in the work-unit could indicate that being highly committed is a health-promoting aspect of work. Indeed, commitment may boost employees’ coping resources [49] and thus alleviate some of the adverse health effects that can emerge from unit-level downsizing. In this way, employees in high-commitment work-units do not experience the same negative health effects as employees in low-commitment work-units.

Noticeably, however, we only found a significant interaction for commitment and unit-level downsizing in the previous quarter for long-term sickness absence, and not for short-term sickness absence. One tentative explanation for this result could be that there is a difference in the length of absence spells between employees in highly committed work-units and employees in units with less commitment to the organization. Put differently, if employees in units with low commitment to the organization not only have more sickness absence spells in the quarter after unit-level downsizing, but their spells also last longer, a higher proportion of their spells becomes long-term.

Finally, we identified a moderating effect of the work-unit level of control on the relationship between unit-level downsizing this quarter and long-term sickness absence. In contrast to earlier research [29, 43], and our expectations, our results show that employees in high-control work-units (15% of the sample) have an increased risk of long-term sickness absence in the change quarter, whereas employees in work-units with less commitment do not. A potential explanation for this result could be that the involuntary nature of unit-level downsizing may be a stronger contrast for employees who otherwise experience a high degree of control. However, the numerous studies supporting the beneficial effects of control and that control did not show as high intergroup agreement as ideally recommended for aggregation [51], suggest that this finding should be interpreted with great caution.

### Strengths and limitations

A strength of this study is the use of several different sources of longitudinal data. The data on organizational change, sickness absence, and moderators were gathered from separate data sources, thus limiting the risk of common-method bias [60]. Moreover, the ability to separate the objective stressor (unit-level downsizing) from employees’ experiences and sickness absence using different measures has been highlighted as an ideal, but not often possible, avenue for investigating stress in organizations [61]. We aggregated data on employees’ perceptions of organizational commitment and control to the work-unit level by using the average degree of perceived commitment and control. Aggregation necessarily means that some data are lost. However, aggregating these data to the work-unit level is also considered as an advantage because it mitigates the possibility of the measures being influenced by individuals’ perceptions, such as general positivity. What is more, aggregated information may be particularly interesting for managers and practitioners, given the focus on group experiences rather than individual differences [62]. Previous research has mainly measured the relationship between organizational change and long-term sickness absence [2, 8, 9]. Thus, another strength of this study is that we also include short-term sickness absence because mechanisms not pertaining to employee health [18, 19] are more likely to influence short-term absence than long-term absence.

The study also has some limitations. The inclusion of survey data rests solely on self-reported measures, which increases the risk of recall bias and other forms of over- and underestimation. However, the QPS Nordic questionnaire seeks to avoid response terminology that carries negative or positive connotations. Specifically, many of the questions ask respondents to report the frequency of an incident as opposed to degrees of satisfaction, seeking to increase the insensitivity to study subjects’ individual dispositions [63]. In this study, the measure of control asks for frequency, whereas the measure for commitment asks for the degree of agreement.

A second limitation concerns the annual nature of the work environment survey. While the work environment variables (control and organizational commitment in the work-units) were measured once per year, our level of analysis was quarterly, meaning that the work environment measures did not necessarily match the quarters of unit-level downsizing. Due to the substantial variation in the relationship between unit-level downsizing and sickness absence from the quarter prior to change to the quarter after, we analyzed the data quarterly. Moreover, the psychosocial work environment rarely remains static throughout a 4-year period. Therefore, it is possible that the degree to which these measures reflect the actual work environment in the respective change quarters is limited. Additionally, the possibility that the self-reported responses reflect the respondents’ experiences with a multitude of stimuli concurrently cannot be completely ruled out [64].

Furthermore, it is important to be aware that earlier research on sickness absence has applied numerous different cut-off points to separate between short- and long-term absence. This needs to be taken into consideration when comparing findings across studies. In this study, we applied a cut-off point of 9 days of sickness absence, representing the length at which a medical sickness absence certificate is required. In this manner, we defined a distinction between short- and long-term sickness absence that is meaningful within the specific research context. Finally, it is important to acknowledge that we have tested multiple possible interactions terms (three moderators x three change quarters x two absence measures). The number of analyses and relevant correlations increased the risk of identifying significant effects by chance (i.e., false significance) [65]. If we had adopted a stricter significance level of *P* < .01, compared to the more common significance level of *P* < .05, to account for multiple testing, only temporary contract would remain as a significant moderator. However, although lowering or adjusting (e.g., Bonferroni corrections) may reduce the risk of Type I errors, these strategies can also increase the risk of Type II errors and thus lessen the statistical power of the analyses [66]. Therefore, based on these arguments, we set the level of significance to *P* < .05.

### Practical implications

Increased knowledge about the ways in which organizational factors may influence absence rates, in either direction, may help practitioners and healthcare managers to make better evidence-based decisions when implementing and facilitating change. Our study, which identifies work-related moderators that may reduce change-related sickness absence and promote employee health, therefore has important practical implications. Working in units with high organizational commitment was related to a steeper decline in short-term sickness absence prior to change and a smaller increase in long-term sickness absence in the period after change. Boosting organizational commitment therefore seems to be a beneficial strategy to increase employees’ aptitude for change. In theory, our results also demonstrate that managers may use temporary employment contracts to reduce change-related sickness absence. However, such strategies raise ethical concerns. From a practical point of view, it is also important to recognize that decreased sickness absence prior to change among temporary employees and employees working in highly committed units likely reflects increased attendance motivation rather than improved health. Therefore, in times of organizational change, sickness absence might be a weaker measure of employee health in organizations with a high proportion of temporary employees. In times of organizational change, it seems particularly important for managers of organizations with a high degree of temporary employees or a very committed employee group to be aware that low sickness absence numbers might not accurately reflect employee health. Therefore, healthcare managers and practitioners should be attentive to employee health and wellbeing, particularly relating to organizational change.

#### Avenues for future research

The current paper has shown that work factors, such as type of employment contract and organizational commitment, may act as important moderators during organizational change, explaining why employees may react differently to similar changes. A workplace, however, affords a wide variety of work environment exposures [67, 68], possibly influencing employees’ change appraisals differently. Therefore, future research may benefit from including a more varied mix of work environment variables as potential moderators, such as job demands or management support. Additionally, future studies should test the potential moderating effects of work environment exposures on various types of change that are often instigated within healthcare organizations, such as mergers [69], acknowledging that what is important during one type of change is not necessarily the same as during a different type of change.

## Conclusion

This study has demonstrated that work-factors interact with different stages of unit-level downsizing to influence the risk of both short- and long-term sickness absence. In particular, the decline in sickness absence prior to unit-level downsizing was stronger for employees on temporary contracts and for employees in work-units with high average scores on organizational commitment. The results thus support that job insecurity is an important disciplinary factor in times of unit-level downsizing, but it is not the sole driver of increased attendance motivation. Moreover, only employees working in units with low organizational commitment had an increase in the risk of long-term sickness absence in the quarter after unit-level downsizing. These results suggest that organizational commitment may be a positive motivator promoting attendance in times of unit-level downsizing as well as contain health-promoting components that lessen the potential negative health consequences often associated with downsizing. Taken together, the identification of temporary contracts and organizational commitment as moderators contributing to an increased or decreased risk of sickness absence constitutes a practical point of departure for managers and policy-makers to promote healthy organizational change.

## Supplementary information

**Additional file 1: Table A.** Associations between unit-level downsizing and sickness absence – moderation effects, including control variables and all moderators.

## Data Availability

The datasets analyzed during the current study are not publicly available. The data are the property of the participating hospital and will therefore not be shared. Upon reasonable request, the corresponding author can facilitate contact with the participating hospital.

## Abbreviations

CI: Confidence interval
EM: Expectation-Maximization algorithm
EUR: Euro
GP: General Practitioner
HR: Human Resources
ID: Identification
IT: Information Technology
NOK: Norwegian kroner
NS: Not significant
NSD: Norwegian Social Science Data Service
OR: Odds ratio
SA: Sickness absence
SD: Standard deviation
