# Supplementary material for: Work-related moderators of the relationship between organizational change and sickness absence: a longitudinal multilevel study
Source: BMC Public Health. 2020 Aug 8;20:1218. doi: 10.1186/s12889-020-09325-w (PMC7414577; doi:10.1186/s12889-020-09325-w)
Supplement: Supplementary file 1 — Additional file 1: Table A. Associations between unit-level downsizing and sickness absence – moderation effects, including control variables and all moderators. [file 12889_2020_9325_MOESM1_ESM.docx]

| Additional File 1, Table A: Associations between unit-level downsizing and sickness absence – moderation effects, including control variables and all moderators | | | | | | | | | | | | | | | | |
| --- | --- | --- | --- | --- | --- | --- | --- | --- | --- | --- | --- | --- | --- | --- | --- | --- |
|  | **Model 1^a^** | | |  |  | | |  | **Model 3^b^** | | |  |  | | |  |
|  | **Short-term SA** | | |  | **Long-term SA** | | |  | **Short-term SA** | | |  | **Long-term SA** | | |  |
|  | (˂=8 days) | | |  | (>=9 days) | | |  | (˂=8 days) | | |  | (>=9 days) | | |  |
|  | **OR** | **95% CI** | |  | **OR** | **95% CI** | |  | **OR** | **95% CI** | |  | **OR** | **95% CI** | |  |
| Unit-level downsizing next quarter | 0,79 | (0,73 | - 0,85) | *** | 0,94 | (0,85 | - 1,04) | ns | 0,97 | (0,62 | - 1,53) | ns | 0,89 | (0,47 | - 1,69) | ns |
| Unit-level downsizing this quarter | 0,99 | (0,92 | - 1,07) | ns | 0,95 | (0,86 | - 1,05) | ns | 1,00 | (0,65 | - 1,54) | ns | 0,51 | (0,27 | - 0,95) | * |
| Unit-level downsizing previous quarter | 1,14 | (1,05 | - 1,24) | *** | 1,05 | (0,94 | - 1,17) | ns | 1,13 | (0,71 | - 1,79) | ns | 2,29 | (1,22 | - 4,30) | * |
| Temporary contract (TC) (scale 0-1) | 0,94 | (0,90 | - 0,98) | *** | 0,58 | (0,55 | - 0,62) | *** | 0,97 | (0,92 | - 1,02) | ns | 0,65 | (0,61 | - 0,70) | *** |
| Unit-level downsizing next quarter X TC | 0,64 | (0,56 | - 0,74) | *** | 0,83 | (0,67 | - 1,03) | ns | 0,78 | (0,65 | - 0,93) | ** | 0,94 | (0,71 | - 1,23) | ns |
| Unit-level downsizing this quarter X TC | 0,99 | (0,86 | - 1,13) | ns | 0,97 | (0,78 | - 1,22) | ns | 1,11 | (0,94 | - 1,32) | ns | 1,20 | (0,92 | - 1,58) | ns |
| Unit-level downsizing previous quarter X TC | 1,03 | (0,87 | - 1,22) | ns | 0,75 | (0,57 | - 1,00) | * | 1,08 | (0,89 | - 1,32) | ns | 0,80 | (0,58 | - 1,12) | ns |
|  |  |  |  |  |  |  |  |  |  |  |  |  |  |  |  |  |
| Unit-level downsizing next quarter | 0,52 | (0,40 | - 0,66) | *** | 0,70 | (0,49 | - 1,01) | ns |  |  |  |  |  |  |  |  |
| Unit-level downsizing this quarter | 1,18 | (0,93 | - 1,50) | ns | 0,61 | (0,43 | - 0,87) | ** |  |  |  |  |  |  |  |  |
| Unit-level downsizing previous quarter | 1,31 | (1,01 | - 1,70) | * | 1,17 | (0,81 | - 1,69) | ns |  |  |  |  |  |  |  |  |
| Control (scale 0-4) | 0,89 | (0,85 | - 0,93) | *** | 0,90 | (0,86 | - 0,95) | *** | 0,92 | (0,88 | - 0,97) | ** | 0,93 | (0,88 | - 0,98) | *** |
| Unit-level downsizing next quarter X control | 1,15 | (1,01 | - 1,32) | * | 1,13 | (0,93 | - 1,37) | ns | 1,11 | (0,97 | - 1,28) | ns | 1,06 | (0,87 | - 1,29) | ns |
| Unit-level downsizing this quarter X control | 0,94 | (0,83 | - 1,07) | ns | 1,28 | (1,06 | - 1,53) | * | 0,96 | (0,84 | - 1,10) | ns | 1,23 | (1,02 | - 1,49) | * |
| Unit-level downsizing previous quarter X control | 0,96 | (0,84 | - 1,10) | ns | 0,94 | (0,78 | - 1,14) | ns | 0,96 | (0,83 | - 1,11) | ns | 0,96 | (0,79 | - 1,18) | ns |
|  |  |  |  |  |  |  |  |  |  |  |  |  |  |  |  |  |
| Unit-level downsizing next quarter | 1,14 | (0,75 | - 1,73) | ns | 0,98 | (0,54 | - 1,77) | ns |  |  |  |  |  |  |  |  |
| Unit-level downsizing this quarter | 0,97 | (0,65 | - 1,46) | ns | 0,64 | (0,36 | - 1,15) | ns |  |  |  |  |  |  |  |  |
| Unit-level downsizing previous quarter | 1,12 | (0,73 | - 1,74) | ns | 2,09 | (1,17 | - 3,75) | * |  |  |  |  |  |  |  |  |
| Organizational commitment (OC) (scale 0-4) | 0,94 | (0,90 | - 0,97) | ** | 0,89 | (0,84 | - 0,94) | *** | 0,95 | (0,91 | - 0,99) | * | 0,88 | (0,83 | - 0,93) | *** |
| Unit-level downsizing next quarter X OC | 0,82 | (0,71 | - 0,96) | * | 0,96 | (0,77 | - 1,18) | ns | 0,84 | (0,72 | - 0,98) | * | 0,98 | (0,79 | - 1,22) | ns |
| Unit-level downsizing this quarter X OC | 1,04 | (0,90 | - 1,19) | ns | 1,15 | (0,94 | - 1,42) | ns | 1,04 | (0,90 | - 1,21) | ns | 1,09 | (0,89 | - 1,35) | ns |
| Unit-level downsizing previous quarter X OC | 1,03 | (0,88 | - 1,20) | ns | 0,78 | (0,63 | - 0,96) | * | 1,05 | (0,90 | - 1,23) | ns | 0,79 | (0,64 | - 0,98) | * |
| Logistic regression. Random effects analyses: random intercept for work-unit. | | | | | | | | | | | | | | | | |
| Abbreviations: SA = sickness absence; OR = odds ratio; CI = 95% confidence intervals; ns = not significant; TC = temporary contracts; OC = organizational commitment | | | | | | | | | | | | | | | | |
| Significance: * p < .05; ** p < .01; *** p < .001. | | | | | | | | | | | | | | | | |
| a Control variables not included | | | | | | | | | | | | | | | | |
| b Control variables, and all interactions included in the same analyses. Control variables include temporary contract, control, organizational commitment, salary, gender, age, multiple job holder, position, nationality | | | | | | | | | | | | | | | | |
| N-work-units: 1,167; N-employees: 21,085; N-observations: 173,787 | | | | | | | | | | | | | | | | |
